# Presepsin as a Prognostic Biomarker for Short-Term Mortality in Adult Patients With Sepsis: A Systematic Review

**DOI:** 10.7759/cureus.107323

**Published:** 2026-04-19

**Authors:** Akshay Rajeev, Sabah Shakeel Shaikh, Harnoor Kaur, Archit Sharma, Palangutde Katherine Dimowo, Bhavna Murugesh

**Affiliations:** 1 Hematology, Great Western Hospital NHS Foundation Trust, Swindon, GBR; 2 Diabetes and Endocrinology, California Institute of Behavioral Neurosciences & Psychology, Fairfield, USA; 3 Internal Medicine, California Institute of Behavioral Neurosciences & Psychology, Fairfield, USA; 4 Pediatrics, Wrightington, Wigan and Leigh Teaching Hospitals NHS Foundation Trust, Wigan, GBR; 5 Otolaryngology - Head and Neck Surgery, California Institute of Behavioral Neurosciences & Psychology, Fairfield, USA; 6 Infectious Disease, Addenbrooke's Hospital, Cambridge University Hospitals NHS Foundation Trust, Cambridge, GBR

**Keywords:** presepsin (cd14-st), scd14, sepsis and septic shock, sepsis markers, sepsis prognosis

## Abstract

Sepsis is a life-threatening medical condition that continues to be a significant global health concern. It is well recognised as a leading cause of morbidity and mortality among hospitalised and critically ill patients of all age groups. Several clinical severity scores, such as the Sequential Organ Failure Assessment (SOFA) and the Acute Physiology and Chronic Health Evaluation II (APACHE), are commonly used to identify and categorise sepsis. However, such clinical tools often require multiple physiological parameters and may not always provide rapid prognostic information. Similarly, inflammatory biomarkers such as C-reactive protein and procalcitonin exhibit variable prognostic value when used alone. Presepsin, a soluble CD14 subtype released when macrophages and monocytes are activated as part of the human innate immune response to infection, has emerged as a promising biomarker to predict infection severity and adverse outcomes in sepsis. This systematic review was conducted to evaluate whether presepsin could serve as a reliable marker to predict short-term (28-day or 30-day) mortality among adult patients with sepsis. A detailed literature search and screening were conducted using PRISMA 2020 (Preferred Reporting Items for Systematic Reviews and Meta-Analyses 2020) guidelines across multiple electronic databases, including PubMed, ScienceDirect, Cochrane Library, and Embase. Following the screening process, a total of 19 observational studies and one systematic review were ultimately selected for the final analysis. Presepsin levels were generally higher in patients with greater disease severity and frequently higher in non-survivors. Although presepsin demonstrated moderate prognostic performance, established clinical severity scores often outperformed presepsin. Overall, existing evidence suggests that presepsin may provide additional prognostic value when integrated with established clinical scoring tools.

## Introduction and background

Sepsis continues to be a major challenge to healthcare systems across the world despite significant medical and global advancements. It is widely recognised as a leading cause of morbidity and mortality among hospitalised and critically ill patients of all age groups globally. Available data indicate that nearly 49 million people are affected by sepsis, leading to 11 million deaths annually [1]. According to the sepsis-3 consensus, sepsis is defined as a life-threatening event that results from organ dysfunction due to a dysregulated host response to infection [2]. Although the awareness among the public regarding sepsis, screening tools for early identification, and treatment guidelines have all improved over time, sepsis-related mortality still remains high, often reported at around 20%-30% in various patient populations and healthcare settings [1,3]. These figures emphasise the necessity for better tools to identify individuals at increased risk and guide treatment strategies.

Early identification of patients who are at high risk of clinical deterioration is crucial for timely escalation of treatment. Several clinical scoring systems are currently in use to evaluate organ dysfunction and illness severity, including the Sequential Organ Failure Assessment (SOFA) [4] and the Acute Physiology and Chronic Health Evaluation II (APACHE II) [5]. Although these tools are helpful for early identification of sepsis and associated organ dysfunction, their effectiveness in rapid prognostic prediction is limited due to the requirement of multiple physiological and biochemical parameters, which may delay timely intervention [6]. Newer rapid bedside tools, such as the quick SOFA (qSOFA), have gained widespread acceptance, but subsequent evidence demonstrates their poor discriminatory ability for predicting in-hospital mortality, thus limiting their reliability as standalone prognostic markers [7]. Similarly, commonly used biomarkers such as C-reactive protein (CRP) and procalcitonin (PCT) are frequently elevated in sepsis. However, they show inconsistent diagnostic and prognostic accuracy across studies when used alone, thus questioning their credibility in clinical practice [8].

Presepsin, also known as the soluble CD14 subtype (sCD14-ST), is a novel biomarker increasingly recognised for its utility in the early detection and prognostication of sepsis. It is generated during the activation of monocytes and macrophages as part of the innate immune response, and its level increases rapidly following bacterial infection. Several studies have reported that high presepsin levels are associated with greater disease severity and poorer outcomes such as organ dysfunction and death [9-11]. However, available literature is variable and at times conflicting. While many studies report high prognostic significance, others demonstrate limited or inconsistent accuracy in predicting prognosis [12,13]. In addition, although presepsin has been evaluated in multiple clinical contexts, its specific ability to predict short-term mortality, particularly 28-day or 30-day mortality, has not been systematically assessed in a recent focused review. Short-term (28-day or 30-day) mortality is a clinically meaningful endpoint in sepsis as it reflects the acute phase during which most sepsis-related deaths occur while minimising the confounding influence of long-term comorbidities.

This systematic review aims to evaluate the role of presepsin in predicting short-term mortality among adult patients with sepsis. By evaluating results from the available literature, this review aims to provide a clearer understanding of its mortality predictive utility, compare its performance with established biomarkers and clinical scoring systems, and determine whether presepsin could serve as a valuable tool in early risk stratification of sepsis in adult clinical practice. In this review, short-term mortality is defined as 28-day or 30-day mortality.

## Review

Methods

PRISMA (Preferred Reporting Items for Systematic Review and Meta-Analyses) 2020 guidelines [14] were followed in conducting this systematic review. The review methods were predefined before conducting the study, and no significant deviations were made.

Search Sources and Strategy

PubMed, ScienceDirect, Cochrane Library, Multidisciplinary Digital Publishing Institute (MDPI), and Embase via Ovid were searched systematically using predefined keywords and Boolean combinations. In PubMed, three different search strategies, including a Medical Subject Headings (MeSH) strategy, were used to maximise the retrieval of relevant articles. A date filter was applied to include studies published within the five-year period from October 10, 2020, to October 10, 2025, across all database searches. This time limit was used to ensure that this review included the most recent and clinically relevant articles that were in line with current practice. The search strategy used for different databases and the number of articles retrieved after each search are summarised in Table 1.

**Table 1:** Summary of different search strategies used across various databases and the number of articles retrieved MDPI: Multidisciplinary Digital Publishing Institute.

Search Strategy	Database	Number of Papers Identified
Sepsis AND Presepsin	PubMed	200
((( "Sepsis/blood"[Mesh] OR "Sepsis/complications"[Mesh] OR "Sepsis/diagnosis"[Mesh] OR "Sepsis/mortality"[Mesh] )) AND ("Sepsis/blood"[Majr] OR "Sepsis/complications"[Majr] OR "Sepsis/diagnosis"[Majr] OR "Sepsis/mortality"[Majr])) AND "presepsin protein, human" [Supplementary Concept]	PubMed	56
(SEPSIS[Title]) AND (PRESEPSIN[Title/Abstract])	PubMed	110
Sepsis AND Presepsin	ScienceDirect	181
Sepsis AND Presepsin	Cochrane Library	17
Sepsis AND Presepsin	MDPI	45
Exp sepsis/PRESEPSIN.mp. 1 and 2 limit 3 to (English language and yr="2020 - Current")	Embase (Ovid)	317

Inclusion and Exclusion Criteria

We included studies published in English that comprised only adult populations aged 18 years or older. To ensure consistency in the assessment of the performance of presepsin, only studies in which presepsin was measured within a clearly defined early time window, preferably within 24 hours of clinical presentation or sepsis diagnosis, were considered. Eligible studies were required to report 28-day or 30-day mortality as an outcome. Only studies published within the previous five years were included.

Studies were excluded if they were narrative reviews or classified as grey literature (e.g., conference abstracts and unpublished data). Research involving paediatric populations was excluded. Studies that failed to clearly mention the timing of presepsin measurement and those evaluating solely the diagnostic performance of presepsin without providing prognostic outcomes were also excluded. Animal studies, laboratory-based in-vitro investigations, and other non-clinical studies were also excluded from the review.

Selection Process

Records retrieved after the search were imported into Zotero. Each article was screened and assessed by two reviewers independently. Initially, duplicates were identified and removed. Later, titles and abstracts were screened to identify and remove irrelevant studies. Full-text articles were then retrieved and evaluated in detail, and articles meeting the inclusion-exclusion criteria were shortlisted.

In instances where uncertainties arose about the inclusion of a study, the issue was reviewed collectively by the co-authors, and eligibility was determined by mutual agreement. Articles without direct full-text availability were retrieved through institutional resources, alternate databases, or direct communication with the corresponding authors. Studies that could not be retrieved despite these efforts were classified as lacking full-text availability and were excluded on this basis.

Quality Assessment of the Studies and Data Extraction

The quality of the shortlisted articles was then assessed using appropriate appraisal tools. The Newcastle-Ottawa Scale (NOS) was used as the appraisal tool for observational studies [15], while systematic reviews were appraised using the AMSTAR-2 (A Measurement Tool to Assess Systematic Reviews, version 2​​​​​) tool [16]. Observational studies were included only if they were of good quality (at least seven stars in NOS), and systematic reviews were included only if they were of moderate or high confidence. Following the final selection of eligible articles, relevant data were extracted and reviewed.

Results

Study Identification and Selection

**Figure 1:**
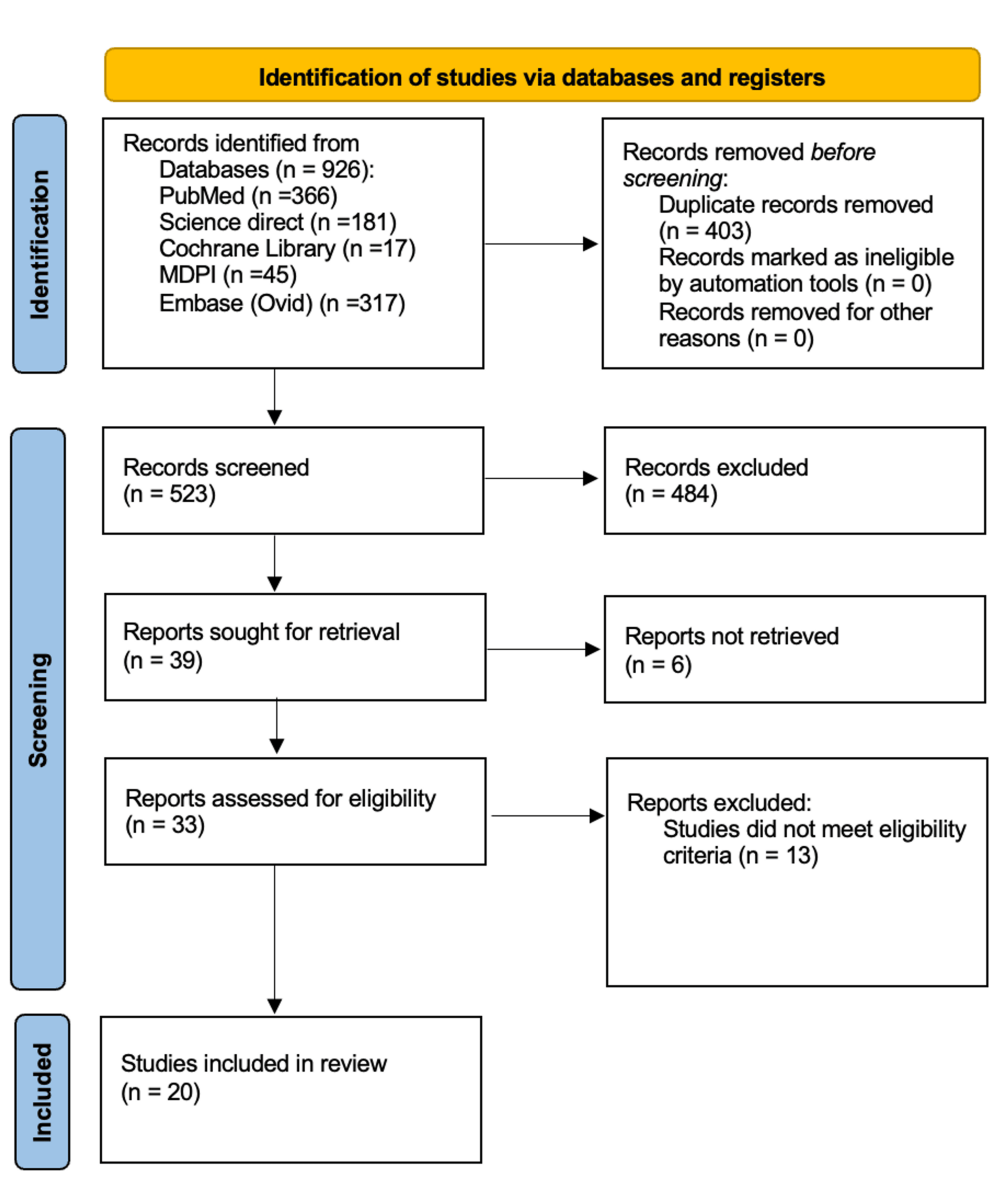
PRISMA flow diagram of study selection PRISMA: Preferred Reporting Items for Systematic Reviews and Meta-Analyses.

Search strategies implemented across all the included databases retrieved a total of 926 studies. During the initial screening of retrieved studies, 403 duplicates were identified and removed, leaving 523 studies for title and abstract screening to determine their relevance to the review. From this screened set, 39 studies appeared suitable for further assessment. Full-text articles were available for 33 studies; the six studies for which full-text versions could not be obtained were excluded from further evaluation. The 33 full-text articles were assessed for eligibility against the inclusion and exclusion criteria. Ultimately, 20 articles met all eligibility requirements and were selected for the systematic review. The final analysis included one systematic review [17] and 19 observational studies [18-36]. The study selection process is summarised as a PRISMA flow diagram in Figure 1.

Quality Assessment

The quality of the included 20 studies was then evaluated using appropriate appraisal tools. The observational studies were assessed using the NOS, and the AMSTAR-2 tool was used for the systematic review. The included observational studies generally demonstrated moderate to good methodological quality, while the systematic review/meta-analysis was rated as moderate confidence according to the AMSTAR-2 criteria. The detailed NOS and AMSTAR-2 appraisals are summarised in Tables 2, 3.

**Table 2:** Newcastle-Ottawa Scale (NOS) quality assessment table NOS: Newcastle-Ottawa Scale. Maximum score = 9 stars.

Author, Year	Number of stars			
	Selection	Comparability	Outcome	Total
Li et al., 2020 [18]	4	0	3	7
Lee et al., 2022 [19]	4	2	3	9
Dobiáš et al., 2022 [20]	4	1	3	8
Roy et al., 2023 [21]	4	0	3	7
Park et al., 2021 [22]	4	2	3	9
Han et al., 2023 [23]	4	1	3	8
Lee et al., 2022 [24]	4	1	3	8
Park et al., 2022 [25]	3	2	3	8
Lee et al., 2024 [26]	4	2	3	9
Iskandar et al., 2025 [27]	4	1	3	8
Kyriazopoulou et al., 2023 [28]	4	2	3	9
Yang et al., 2024 [29]	4	1	3	8
Kim et al., 2023 [30]	4	1	3	8
Shimoyama et al., 2021 [31]	4	2	3	9
Ozkan et al., 2021 [32]	4	1	3	8
Kahveci et al., 2021 [33]	4	1	3	8
Wu et al., 2023 [34]	4	1	2	7
Koh et al., 2021 [35]	4	1	3	8
Wang et al., 2020 [36]	4	0	3	7

**Table 3:** AMSTAR-2 quality appraisal of the included systematic review The literature search was considered partial because grey literature and trial registries were not included. RoB: Risk of bias; AMSTAR-2: A Measurement Tool to Assess Systematic Reviews, version 2; PICO: Population, intervention, comparison, and outcome.

AMSTAR-2 Criteria	Molano-Franco et al., 2023 [17]
PICO components included	Yes
Protocol established prior to review	Yes
Justification of study design selection	Yes
Comprehensive literature search	Partial Yes
Study selection in duplicate	Yes
Data extraction in duplicate	Yes
List of excluded studies provided	Yes
Adequate description of included studies	Yes
Risk of bias assessment	Yes
Reporting of funding sources	Yes
Appropriate meta-analysis methods	Yes
Impact of RoB assessed	Yes
RoB considered in the interpretation	Yes
Explanation of heterogeneity	Yes
Publication bias assessed	Yes
Conflict of interest reported	Yes

Outcomes Measured

The primary outcome measured was 28-day or 30-day mortality following a sepsis diagnosis, presentation to the emergency department, or ICU admission. Secondary outcomes frequently included severity scores for organ dysfunction (SOFA, APACHE, and qSOFA), need for vasopressors, and presepsin dynamics with treatment or disease progression. Several studies also examined associations between presepsin and parameters such as monocyte human leukocyte antigen-DR (HLA‑DR), programmed death-ligand 1 (PD‑L1) expression, and blood culture positivity [18,21,27]. One study also looked into the ability of presepsin to distinguish sepsis due to fungal versus bacterial infection [20].

Study Characteristics

The observational studies included 4785 adults with suspected sepsis and 326 healthy controls. The systematic review and meta-analysis involved 60 studies comprising 15,681 critically ill septic adults. The observational studies were carried out in hospitals across China, South Korea, Turkey, Japan, India, the Czech Republic, Greece, and Indonesia, encompassing patients in emergency departments, intensive care units, and acute wards. Three of the observational studies were multicentric [20,28,33], while the others included only a single centre. Several studies focused on specific scenarios such as urinary tract infections [29], community-acquired pneumonia [32], fungal sepsis [20], and renal impairment [23,26,30]. Most studies used Sepsis-3 criteria or closely aligned definitions for sepsis diagnosis and severity classification. Study characteristics are summarised in Table 4.

**Table 4:** Summary of study characteristics of the included studies PCT: Procalcitonin; CD14: Cluster of differentiation 14; HLA-DR: Human leukocyte antigen-DR (isotype); SOFA: Sequential Organ Failure Assessment; APACHE II: Acute Physiology and Chronic Health Evaluation II; CRP: C-reactive protein; IL-6: Interleukin-6; SAPS: Simplified acute physiology score; ED: Emergency department; (1,3)-β-D-glucan: 1,3 beta-D-glucan; AKI: Acute kidney injury; CRRT: Continuous renal replacement therapy; NEWS: National early warning score; MEWS: Modified early warning score; qSOFA: Quick Sequential Organ Failure Assessment; WCC: White cell count; UTI: Urinary tract infection; ICU: Intensive care unit; PNI: Prognostic nutritional index; PSI: Pneumonia severity index; MEDS: Mortality in emergency department sepsis score; Hb: Haemoglobin; Urea: Blood urea; APTT: Activated partial thromboplastin time; AST: Aspartate aminotransferase.

Author (Year)	Study Design	Sample Size	Purpose of the Study	Comparator Biomarkers/Scores	Results	Conclusion
Iskandar et al. (2025) [27]	Prospective single-centre observational cohort study	110	Evaluate the prognostic value of presepsin for short-term mortality	PCT, SOFA, and culture status	Presepsin remained independently associated with mortality	May support early clinical decision-making
Yang et al. (2024) [29]	Prospective observational single-centre study	171	Predict septic shock and mortality in UTI	Lactate, PCT, CRP, and WCC	Superior to CRP and PCT; comparable to lactate for shock prediction	Provides prognostic information in urosepsis
Lee et al. (2024) [26]	Retrospective observational single-centre study	57	Prognostic value in AKI requiring CRRT	APACHE II, SOFA, CRP, PCT, and lactate	Better discrimination than APACHE II, SOFA, CRP, and PCT in sepsis-associated AKI	Limited in the general CRRT population but useful in sepsis-associated AKI
Roy et al. (2023) [21]	Prospective observational single-centre study	82	Compare diagnostic and prognostic performance vs PCT	PCT and culture positivity	Higher presepsin associated with increased mortality risk	Demonstrates prognostic relevance
Molano-Franco et al. (2023) [17]	Systematic review and meta-analysis	60 studies (15,681 adults)	Evaluate prognostic value for mortality	PCT, CRP, IL-6, SOFA, APACHE, and SAPS	Minimal effect on 28-day mortality	Limited independent prognostic value
Han et al. (2023) [23]	Retrospective observational single-centre study	127	Prognostic utility in severe AKI with CRRT	CRP, PCT, SOFA, and lactate	Not independently associated with mortality	Less reliable than CRP or PCT
Kyriazopoulou et al. (2023) [28]	Prospective derivation and validation cohort study	345	Diagnostic and prognostic stratification	CRP, PCT, WCC, APACHE II, SOFA, and qSOFA	Outperformed CRP and WCC; comparable/superior to PCT	Provides independent diagnostic and prognostic value
Kim et al. (2023) [30]	Retrospective observational single-centre study	420	Prognostic performance in organ failure with/without hypercreatinaemia	PCT, CRP, and SOFA	Better discrimination than CRP and PCT	Useful when renal function is considered
Wu et al. (2023) [34]	Single-centre case-control study with machine-learning analysis	467	Diagnostic and prognostic evaluation	CRP, PCT, Hb, urea, APTT, and AST	Comparable to PCT; superior to CRP; improved with combined models	Enhanced value when combined with laboratory parameters
Lee et al. (2022) [19]	Retrospective observational single-centre study	249	Prognostic utility in ED	PCT, CRP, lactate, APACHE II, and SAPS III	Not independently associated with mortality	Limited prognostic value
Dobiáš et al. (2022) [20]	Multicentre retrospective observational cohort study	165	Diagnosis and prognosis in Candida sepsis	(1,3)-β-D-glucan, CRP, PCT, SOFA, and APACHE II	Stronger association with mortality than PCT	Useful in fungal sepsis
Lee et al. (2022) [24]	Prospective observational single-centre study	420	Differentiate infectious vs non-infectious organ failure	PCT, CRP, lactate, SOFA, APACHE II, NEWS, and MEWS	Independently associated with mortality	Useful diagnostic and prognostic tool
Park et al. (2021) [22]	Retrospective cross-sectional single-centre study	757	Predict infection and mortality	PCT, CRP, qSOFA, and Charlson index	Superior prognostic performance	Useful for short-term mortality prediction
Shimoyama et al. (2021) [31]	Prospective observational single-centre pilot study	83	Predict mortality and compare with inflammatory scores	PNI, SOFA, and qSOFA	Independently associated with mortality; superior to inflammatory scores	Enhances risk stratification
Ozkan et al. (2021) [32]	Prospective observational single-centre study	176	Prognostic value in pneumonia-related sepsis	PCT, CRP, lactate, PSI, and qSOFA	Associated but not superior to existing markers	Limited added prognostic value
Park et al. (2022) [25]	Prospective observational single-centre study	755	Identify high-risk patients	SOFA, lactate, and PCT	Better discrimination than PCT	Improves early risk identification
Kahveci et al. (2021) [33]	Prospective observational multicentre study	150	Predict septic shock and mortality	PCT, CRP, MEDS, and qSOFA	No independent association	No advantage over conventional markers
Koh et al. (2021) [35]	Retrospective observational single-centre cohort study	153	Prognostic value	Lactate, CRP, PCT, APACHE II, and SOFA	Associated with mortality; inferior to SOFA	Limited prognostic accuracy
Li et al. (2020) [18]	Observational single-centre cohort study	238	Association with immune dysfunction and outcomes	PCT, CD14, HLA-DR, PD, SOFA, and APACHE II	Associated with severity and mortality	Useful adjunct biomarker
Wang et al. (2020) [36]	Prospective observational single-centre study	186	Diagnostic and prognostic value	PCT, CRP, IL-6, APACHE II, and SOFA	Associated with mortality but not superior	Limited prognostic value

For the measurement of presepsin, most studies used an automated chemiluminescent enzyme immunoassay (CLEIA) on the PATHFAST analyser using plasma or whole blood, while only a minority used other methods like ELISA [21].

In general, presepsin values were highest in septic shock, followed by sepsis, and were lower in non-infective organ failure. Several studies suggested that elevated presepsin was associated with higher 28-day or 30-day mortality, with the area under the ROC curve (AUC) typically between 0.64 and 0.84, and presepsin cut-offs around 700-1200 pg/mL. In multivariable analysis, presepsin often proved to be an independent predictor of short-term mortality, but its independent value in comparison to current clinical scores was modest.

However, the meta-analysis included in this review suggested that isolated presepsin measurement did not significantly help in predicting mortality but could be used as an adjunct to clinical scoring systems [17].

Discussion

This systematic review evaluated the role of presepsin in predicting short-term (28-day or 30-day) mortality in adult patients with sepsis. Analysis of data from the 20 included articles suggests that presepsin levels are generally elevated in non-survivors and exhibited moderate discriminative performance across various clinical settings. However, results varied substantially according to population, renal function, source of infection, and the analytic approach used. Overall, presepsin appears to have greater prognostic value when interpreted in combination with clinical severity scores and other biomarkers.

Pathophysiology of Presepsin

Presepsin, also known as soluble CD14 subtype (sCD14-ST), is a 13-kDa fragment of the CD14 receptor produced during the body’s innate immune response to infection. CD14 is a receptor found on the surface of immune cells such as monocytes, macrophages, and neutrophils. It helps in the pattern recognition of bacterial components, particularly those of bacterial cell walls, by forming complexes with lipopolysaccharide-binding protein (LBP) and transferring these ligands to their appropriate receptors. During phagocytosis of microorganisms, CD14 is cleaved, and a smaller soluble portion gets released into the blood [37].

**Figure 2:**
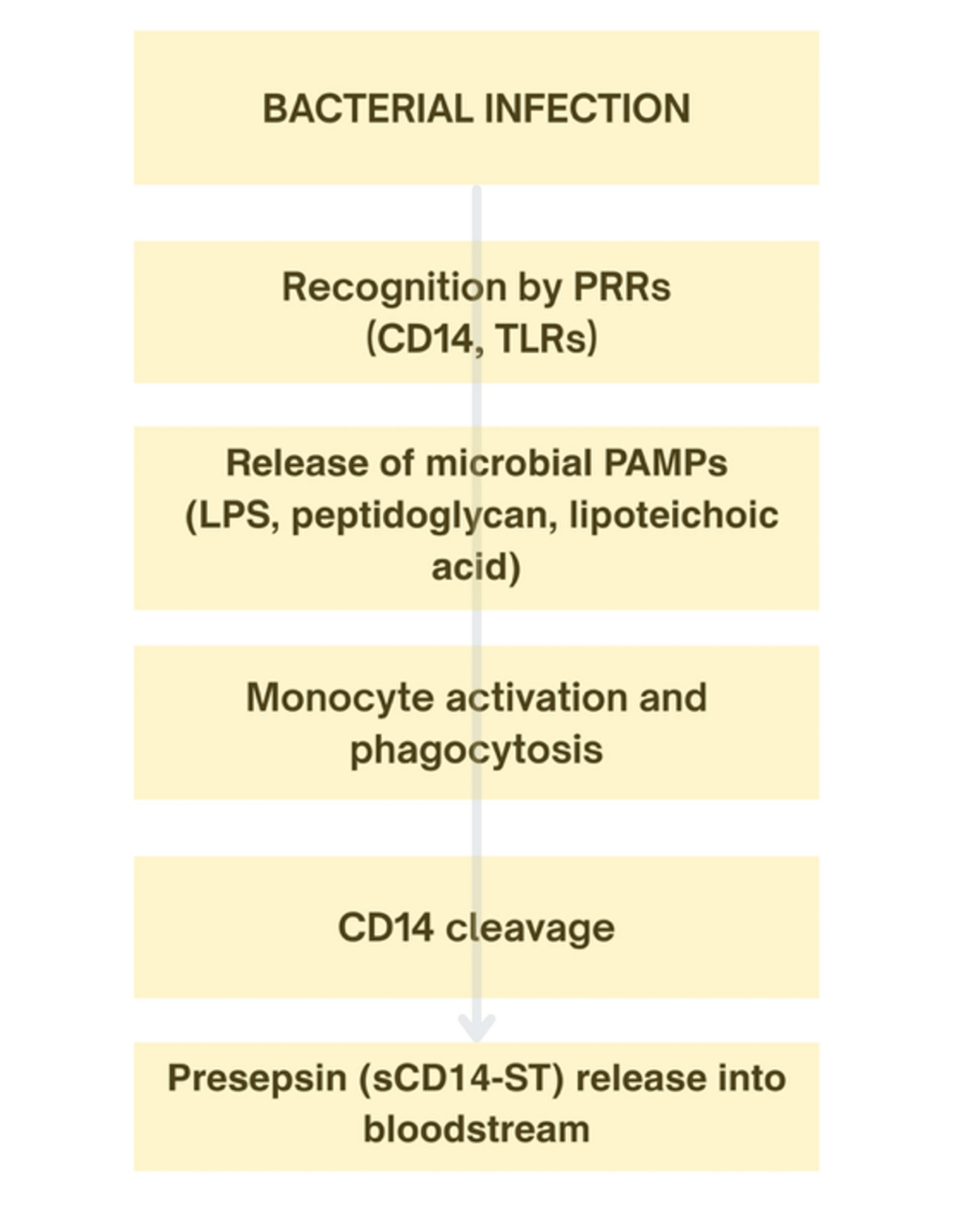
Pathophysiology of presepsin formation during bacterial infection Pathogen-associated molecular patterns (PAMPs), including lipopolysaccharide (LPS), peptidoglycan, and lipoteichoic acid, are recognised by pattern recognition receptors (PRRs) such as CD14 and toll-like receptors (TLRs). This leads to monocyte activation and phagocytosis. During this process, CD14 undergoes proteolytic cleavage, resulting in the release of presepsin (soluble CD14 subtype, sCD14-ST) into the bloodstream. This figure was created by the first author using Canva (Canva Pty Ltd., Sydney, Australia).

In sepsis, presepsin rises early, and its level increases with increasing severity of the innate immune response. Thus, presepsin becomes useful not only in the early diagnosis of sepsis but also in the assessment of disease​​​ severity. Presepsin levels tend to be significantly higher in patients with severe sepsis and septic shock in comparison with those with less severe infection or non-infectious systemic inflammatory response syndrome (SIRS) [37,38]. The key steps involved in the generation of presepsin during bacterial infection are illustrated in Figure 2.

Overall Prognostic Performance Across Studies

Higher presepsin levels were observed among non-survivors in most studies. In the emergency department-based study by Park et al., presepsin demonstrated an AUC near 0.75 for 28-day mortality and remained an independent predictor of mortality even after adjustment of confounders [22]. Similarly, the large cross-sectional study by Park et al. found that presepsin predicted 28-day mortality better than procalcitonin or C-reactive protein [25]. Studies by Dobiáš et al. and Roy et al. also suggested similar positive prognostic abilities of presepsin, especially in critically ill ICU patients [20,21]. Shimoyama et al. showed that day one presepsin independently predicted 28-day mortality in septic ICU patients; the AUC exceeded that of several existing scores, and presepsin retained significance in multivariable analysis [31].

In sepsis due to community-acquired pneumonia, Ozkan et al. found that presepsin levels increased proportionately with disease severity and were higher in non-survivors but did not outperform existing scores such as qSOFA and biomarkers such as CRP, PCT, and lactate in mortality prediction [32]. Yang et al. reported that presepsin levels were independently associated with short-term mortality and that its prognostic utility was better than CRP and PCT and comparable to lactate [29]. In Candida sepsis, Dobiáš et al. found higher presepsin levels in candidemia than in bacteraemia and reported that presepsin performed its role better than PCT in both diagnosis and mortality prediction [20].

However, the systematic review and meta-analysis conducted by Molano-Franco et al. found minimal independent mortality prediction ability of presepsin, but results should be interpreted cautiously given the limited number of sCD14 assessments and variability in adjustments for key confounders and mortality endpoints [17]. The multicentre observational study by Kahveci et al. involving patients attending emergency departments found no significant differences in presepsin between sepsis and septic shock and between survivors and non-survivors [33]. Overall, these findings suggest that although presepsin often proves to be a useful prognostic marker for 28-day mortality, its performance remains variable across populations and study designs.

Comparison With Existing Clinical Scores and Biomarkers

Several studies suggest that presepsin is comparable to and, in many instances, superior to existing biomarkers such as PCT and CRP for predicting 28-day or 30-day mortality. Park et al. found that in patients with sepsis presenting to the emergency department, presepsin showed better accuracy for predicting mortality than PCT, and its accuracy improved when combined with SOFA or lactate [22]. In a recent prospective cohort study, Iskandar et al. observed that presepsin performed similarly to PCT in predicting short-term mortality, and it remained significant irrespective of culture status [27]. In a similar pattern, Li et al. found that presepsin had a significantly higher AUC than PCT for 28-day mortality prediction [18].

Conversely, multiple studies indicate that existing clinical scores outperform presepsin in their ability to predict short-term mortality. Shimoyama et al. found that presepsin was not superior to SOFA on multivariable analysis and suggested that presepsin should be used to complement existing clinical scores rather than replace them. In an observational study in the ICU by Koh et al., presepsin was independently associated with in-hospital mortality, but its AUC was lower than that of SOFA and was only similar to lactate. These findings support the conclusion of Molano-Franco et al. that baseline presepsin has limited prognostic value over existing clinical scores [17,31,35].

In addition, some studies specifically explored the prognostic capability of presepsin when used in combination with other scores and biomarkers. Li et al. found that the combination of presepsin with monocyte HLA-DR mean fluorescence intensity (MFI) yielded the highest AUC for 28-day mortality. The machine-learning analysis by Wu et al. showed that models combining presepsin with routine biomarkers such as haemoglobin, renal function, and coagulation markers achieved better mortality prediction utility than PCT or presepsin alone. Similarly, Park et al. and Lee et al. suggested that presepsin has better prognostic utility when combined with SOFA and lactate, supporting a complementary rather than substitution role [18,22,24,34].

Presepsin and Renal Dysfunction

Renal function significantly influences the prognostic ability of presepsin. As presepsin is partly cleared by the kidneys, its level increases in chronic kidney disease and acute kidney injury (AKI), which can complicate its interpretation. Kim et al. conducted an observational study of 420 patients with organ failure, with and without hypercreatinaemia, and found that although presepsin levels were high in patients with hypercreatinaemia, it showed better prognostic capability than CRP and PCT even in patients with impaired renal function [30].

The study by Lee et al., focusing on patients receiving continuous renal replacement therapy, found that presepsin performed worse than clinical scores such as APACHE II and SOFA for the prediction of 28-day or 30-day mortality. However, in the subgroup with sepsis-induced AKI, presepsin proved to have stronger mortality prediction accuracy than APACHE II, SOFA, CRP, and PCT [26]. This suggests that in patients with sepsis complicated by AKI, presepsin has significant prognostic value and could complement clinical scores.

Hence, renal dysfunction could make the interpretation of presepsin values difficult. Cut-off values for risk stratification are likely to be higher with chronic kidney disease or AKI and should be carefully interpreted.

Clinical Implications

Presepsin is an attractive biomarker, a soluble subtype of CD14 that reflects activation of the innate immune system, is a marker of infection severity and immune dysregulation, and has an association with organ dysfunction and coagulopathy [31]. From a clinical perspective, this review adds that presepsin is higher in septic patients and in non-survivors compared with survivors and is mostly independently associated with 28-day or 30-day mortality [18,22,29,31]. Prognostic accuracy is generally moderate (AUC ~ 0.60-0.75) and, in most settings, does not outperform well-validated clinical scores such as SOFA, APACHE II, or PSI [32,35]. It often performs comparably to or sometimes better than inflammatory biomarkers such as PCT and CRP for mortality prediction [22,27,30]. Presepsin also appears to be promising in specific subgroups, including invasive candidiasis [20], urosepsis [29], and sepsis-associated AKI patients on continuous renal replacement therapy [26]. Studies that evaluated the prognostic ability of serial presepsin measurements found that persistently elevated or non-decreasing levels were more strongly associated with short-term mortality than single baseline values, suggesting that dynamic monitoring may provide superior prognostic information [20,21,31].

Another important clinical value noted across the studies was the wide variation in presepsin cut-off values used to predict 28-day or 30-day mortality. Thresholds used ranged from approximately 500-800 pg/ml in emergency department-based studies and mixed population-based studies [22,24] to over 1200-2000 pg/ml in patients with septic shock or high disease severity and even >10,000 pg/ml in some ICU studies with high illness severity [27]. Studies involving patients with renal failure also reported elevated presepsin cut-offs, reflecting impaired renal clearance. Kim et al. further demonstrated that presepsin values increased proportionately with hypercreatinaemia, suggesting that cut-off values should be adjusted on the basis of the involved population and renal function [30]. Collectively, these findings indicate that there is likely no universally applicable prognostic cut-off for presepsin, and the use of a single threshold risks misinterpretation. Ideally, presepsin appears to have the best utility when interpreted within the clinical context, taking into account disease severity and renal function, and when used in combination with existing clinical scores. This would benefit the use of an immune activation dimension not usually captured by physiology-based scores alone.

Strengths and Limitations

A major strength of this review is that it focused on the role of presepsin specifically for 28-day or 30-day mortality, which represents the most widely accepted short-term outcome measure in sepsis research. By filtering inclusion to studies reporting mortality during this time period, we avoided pooling heterogeneous outcomes such as in-hospital mortality or ICU mortality, thereby improving clinical interpretability. Another strength is the inclusion of a broad range of clinical settings, including emergency departments, ICU patients, disease-specific studies, and patients with renal impairment and renal replacement therapy. Furthermore, several included studies used multivariable analysis and direct comparison against established biomarkers and severity scores, thus providing meaningful comparisons. Finally, the review provided future directions of research and suggestions regarding its clinical utility.

This review also has limitations that need to be acknowledged. Most studies included were single-centre observational studies with relatively small sample sizes, thus limiting the generalisability of the findings. Although most studies adopt Sepsis-3 as the definition for sepsis, few studies use earlier criteria and clinical suspicion of sepsis as inclusion criteria, and presepsin assays and cut-offs used differ widely across studies. This limits the contribution of this review in terms of clinical translation of presepsin cut-off values to predict short-term mortality. Several individual studies adjusted only for a limited set of variables and may not fully account for associated comorbidities, and many studies either do not adjust for renal function or treat it only crudely, despite clear evidence of the association of presepsin and altered renal function. Although presepsin proved to have significant prognostic associations, no study evaluated presepsin-guided clinical decision-making. It remains unknown whether earlier treatment escalation, ICU transfer, or changing antibiotics based on presepsin could improve clinical outcomes. Finally, we acknowledge the possibility of publication bias, as this review included only studies published in English in the last five years​​​​​​ and excluded grey literature, with the probability of missing significantly relevant articles that did not meet the inclusion criteria.

Future Research Directions

Current available data suggest that presepsin remains prognostically interesting but operationally under-tested. Studies evaluating presepsin-guided treatment guidelines remain minimal, and bridging this evidence gap requires further trials and studies. In addition, there is a clear need for large, multicentre studies without global restrictions, universal assays, and the evaluation of serial presepsin kinetics. Carefully designed protocols examining day-to-day trends and their correlation with treatment response could clarify whether presepsin could function as a real-time indicator of immune dysregulation and therapeutic response.

## Conclusions

In conclusion, this review of the 20 included studies suggests that presepsin is a clinically promising adjunctive biomarker for predicting short-term mortality (28-day or 30-day) in adult patients with sepsis. Evidence from the included studies suggests that elevated presepsin levels are generally associated with adverse outcomes. However, the lack of studies evaluating treatment decisions based on presepsin, variability in thresholds, and the impact of renal dysfunction limits its widespread immediate use as an independent marker of prognosis in sepsis. At present, presepsin is best used as an adjunct biomarker to existing clinical scores to improve risk stratification. Further high-quality, multicentre studies are required to clarify its role and determine whether it can be integrated into routine sepsis monitoring and management.
